# Skeletal muscle regeneration in *Xenopus *tadpoles and zebrafish larvae

**DOI:** 10.1186/1471-213X-12-9

**Published:** 2012-02-27

**Authors:** Alexandre Miguel Cavaco Rodrigues, Bea Christen, Mercé Martí, Juan Carlos Izpisúa Belmonte

**Affiliations:** 1 Center for Regenerative Medicine of Barcelona, 08003 Barcelona, Spain; 2 Gene Expression Laboratory, The Salk Institute for Biological Studies, La Jolla, CA 92037, USA

## Abstract

**Background:**

Mammals are not able to restore lost appendages, while many amphibians are. One important question about epimorphic regeneration is related to the origin of the new tissues and whether they come from mature cells via dedifferentiation and/or from stem cells. Several studies in urodele amphibians (salamanders) indicate that, after limb or tail amputation, the multinucleated muscle fibres do dedifferentiate by fragmentation and proliferation, thereby contributing to the regenerate. In *Xenopus laevis *tadpoles, however, it was shown that muscle fibres do not contribute directly to the tail regenerate. We set out to study whether dedifferentiation was present during muscle regeneration of the tadpole limb and zebrafish larval tail, mainly by cell tracing and histological observations.

**Results:**

Cell tracing and histological observations indicate that zebrafish tail muscle do not dedifferentiate during regeneration. Technical limitations did not allow us to trace tadpole limb cells, nevertheless we observed no signs of dedifferentiation histologically. However, ultrastructural and gene expression analysis of regenerating muscle in tadpole tail revealed an unexpected dedifferentiation phenotype. Further histological studies showed that dedifferentiating tail fibres did not enter the cell cycle and *in vivo *cell tracing revealed no evidences of muscle fibre fragmentation. In addition, our results indicate that this incomplete dedifferentiation was initiated by the retraction of muscle fibres.

**Conclusions:**

Our results show that complete skeletal muscle dedifferentiation is less common than expected in lower vertebrates. In addition, the discovery of incomplete dedifferentiation in muscle fibres of the tadpole tail stresses the importance of coupling histological studies with *in vivo *cell tracing experiments to better understand the regenerative mechanisms.

## Background

While all vertebrates are able to repair certain injured tissues and organs, like muscle and liver, only lower vertebrates retain the ability to regrow complex appendages like fins, tails and limbs after amputation. An immense research effort has been made into uncovering the differences behind the disparity in regeneration ability between lower, regenerating vertebrates, and the non-regenerating, higher vertebrates. One of the main differences resides in the formation of the blastema, a structure composed of highly proliferative progenitors that will give rise to the new tissues. Mammals generally fail to form this structure [1-3]. In amphibians and fish, blastema formation happens right after wound healing and relies on the dedifferentiation of mature cells [4-6] and/or the activation of adult stem cells [7-10]. Dedifferentiation, or loss of differentiation characteristics followed by the acquisition of progenitor features like proliferation, was believed to provide cells with the capacity to re-differentiate into different cell lineages [11-13]. However, the blastema was recently shown to mainly contain lineage-restricted progenitors that only give rise to the same kind of tissues from which they originated [6,8,14-16], indicating that dedifferentiation is less extensive than previously thought [13,17,18].

One of the main tissues that contributes to the blastema is the skeletal muscle [4,8,10,14]. Skeletal muscle is mainly composed of elongated cells called muscle fibres, or myofibres, that contract upon nervous stimuli. Muscle fibres are formed during development or regeneration by the fusion of multiple myogenic progenitor cells called myoblasts. For this reason, myofibres are multinucleated, or syncytial, cells. In addition, muscle fibres are also characterized by a highly organized internal structure, necessary for proper contraction of the muscle. The building blocks of this internal structure are the sarcomeres, and the tandem repetition of sarcomeres gives rise to long chains called myofibrils. In turn, several myofibrils are packed inside the muscle fibre with the sarcomeres perfectly aligned, which creates the characteristic striated pattern of the muscle.

Interestingly, early studies in urodeles (salamanders) showed that the striated pattern of limb muscle is lost after amputation [19,20], indicating muscle dedifferentiation. Further studies strongly suggested that limb and tail muscle fibres were able to fragment and that the resulting mononuclear cells proliferate and contribute to the blastema [4,21-23]. Along with muscle dedifferentiation, muscle stem cells (satellite cells) were also shown to contribute to the new muscle [7,10,14]. On the other hand, studies on *Xenopus *tail regeneration revealed that muscle fibres do not contribute to the blastema, indicating the absence of muscle dedifferentiation [8]. Instead, muscle satellite cells were shown to be the main contributor to the new tail muscle [8,9].

Less is known on how the skeletal muscle is regenerated in *Xenopus *tadpole limbs following amputation. Furthermore, there is scarce literature on fish muscle regeneration, but the general belief is that fish, like urodeles, regenerate their tissues by dedifferentiation [5,15,24]. We therefore investigated whether *Xenopus *limb and tail skeletal muscle share the same regenerative strategy (absence of dedifferentiation) and whether zebrafish larvae actually regenerate muscle by dedifferentiation. We opted for two main techniques: *in vivo *tracing of genetically labelled myofibres to see if they dedifferentiated/fragmented and contributed to the new muscle; and histological and gene expression analysis for the detection of dedifferentiation phenotypes. We found that myofibers from the zebrafish tail and the tadpole limb do not dedifferentiate after amputation. On contrary, histological and gene expression studies revealed an unexpected dedifferentiation phenotype in tadpole tail myofibres, similar to what was described in urodeles [19,20,25-28]. However, further *in vivo *studies indicated that the histological dedifferentiation phenotype was associated with myofibre retraction and was not resulting in fragmentation of the fibres.

## Results

### Genetic labelling of muscle fibres for the *in vivo *detection of dedifferentiation

**Figure 1:**
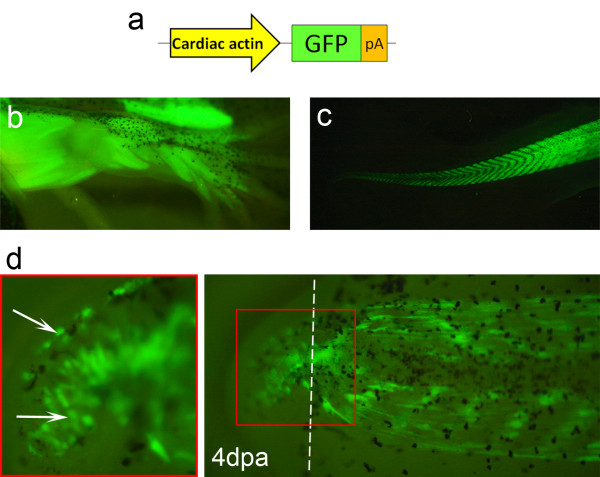
**Alpha-cardiac actin promoter drives muscle specific expression**. (**a**) Representation of the Car-GFP construct, where the muscle specific "alpha-cardiac actin" promoter (Car) is driving the expression of GFP. (**b**, **c**) Car-GFP F_0 _transgenic animals generally show specific and strong GFP expression in muscle fibres of the hindlimbs (**b**) and the tail (**c**). *n *= 25 animals. (**d**) At 4 dpa, many GFP^+ ^cells are observed in the tail regenerate of a Car-GFP F_0 _transgenic tadpole (arrows). Dashed line: amputation plane.

While we have a fair understanding of how muscle regenerates in the *Xenopus *tadpole tail, there is a lack of knowledge about tadpole limb muscle regeneration. To obtain more information on this process, specifically whether limb muscle fibres dedifferentiate/fragment and contribute to the new muscle, we attempted to genetically label limb muscle fibres with GFP and to follow them during regeneration. We first confirmed that the alpha-cardiac actin (Car) promoter was able to drive strong and specific GFP expression in muscle fibres of the limb and tail (Figure 1a-c) [29]. However, these simple Car-GFP transgenics are not useful for cell tracing experiments, since the unlabeled myogenic progenitors activate GFP expression during regeneration and become indistinguishable from any possible unicellular fragments derived from labelled dedifferentiating myofibres (Figure 1d, arrows) [8].

**Figure 2:**
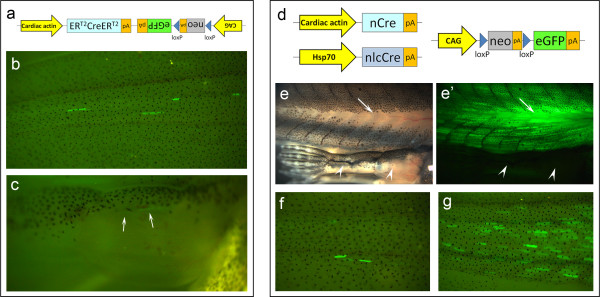
**Inducible Cre constructs do not label limb muscle**. (**a**) Representation of the Car-ERCreER/CALNL-GFP construct, where Car promoter is driving the expression of the tamoxifen-inducible Cre recombinase. On the same vector but in opposite direction, we placed a reporter construct composed of the constitutive CAG promoter driving the expression of a floxed *neo *gene. Downstream of it, there is the *eGFP *gene that is only expressed when Cre is able to remove the floxed *neo *gene. (**b**, **c**) After two weeks of daily intraperitoneal tamoxifen injections, only few myofibres were generally labelled in the tails (**b**) and less than 1% of transgenics had any label in the hindlimb (**c**) of Car-ERCreER/CALNL-GFP F_1 _and F_2 _transgenics (arrows: faint GFP^+ ^myofibres). *n *> 100 animals. (**d**) Representation of the Car-nCre, Hsp-nlcCre and CALNL-GFP constructs. Here, the Cre recombinase is split in two inactive fragments (nCre and nlcCre). In this system, Cre is only active when both fragments are co-expressed. To make the system inducible and muscle-specific, we cloned the nCre fragment under the Car promoter and the nlcCre fragment under a heat-shock-inducible promoter (Hsp70). (**e-g**) After heat-shock treatments of F_1 _tadpoles, we generally observed strong expression of GFP in the trunk (arrow) but no expression in the hindlimbs (compare *e *with *e'*. Arrowheads point to the unlabelled hindlimb). We also observed that the number of labelled myofibres was lower in the middle of the tail (**g**) and even lower closer to the tip of the tail (**f**). *n *> 100 animals.

To avoid myogenic progenitors from getting labelled *de novo *during regeneration and to unambiguously identify myofibre fragments, an inducible labelling system was needed. We used the Cre-lox recombination system, where Cre recombinase can remove (or invert) genomic sequences lying between two *loxP *sites [30]. This allows constitutive labelling of any desired cell lineage when using a tissue specific promoter driving *cre *expression and a ubiquitous promoter followed by lox-STOP-lox and a fluorescent protein gene like GFP. To make this system inducible and to avoid Cre (and GFP) activation during *de novo *myogenesis, we used the well established tamoxifen-inducible Cre system, where Cre is fused to two mutated estrogen receptors (ER^T2^) that inhibit Cre activity in the absence of tamoxifen [31,32]. We, therefore, constructed the "Car-ERCreER/CALNL-GFP" vector containing a tamoxifen-inducible Cre under the control of the Car promoter and the lox-*neo*-lox-*GFP *cassette under control of the constitutive CAG promoter [32,33] (Figure 2a). After Cre induction in founder animals, all we ever achieved were a few randomly labelled muscle fibres in the tail without any positive fibres in the limb. Similar results were obtained with F_1 _and F_2 _offspring from two different transgenic lines, even after two weeks of daily intraperitoneal injections of tamoxifen. We observed very few and dispersed labelled myofibres in the tail (Figure 2b) and, more importantly, only one animal (from hundreds) had three faintly labelled fibres in the limb (Figure 2c, arrows), which did not allow us to study limb regeneration with these transgenics.

As an alternative to the ER^T2^CreER^T2 ^system we tested the alpha complementation of Cre subunits. This system is based on the fact that the Cre protein can be split into two inactive fragments that, when co-expressed, re-associate and restore Cre activity [34]. For higher re-association efficiency, Cre fragments can be fused with dimerizing peptides [35,36]. We reasoned that putting one fragment under the control of an inducible promoter (e.g. the Heat-shock promoter Hsp70) and the other under the Car promoter, the result would be the desired inducible and muscle-specific expression of Cre recombinase. Therefore, we built two new constructs (Figure 2d), one containing the N-terminal Cre fragment (nCre) under the control of the Car promoter and the second with the C-terminal Cre fragment (nlcCre) under the control of the Hsp70 promoter. We injected these two constructs together with the reporter construct, pCALNL-GFP, into *Xenopus *eggs to obtain triple transgenics. After heat-shock induction, some founder transgenics had many labelled tail muscle fibres, but still no labelled limb fibres. Similarly, the offspring of a founder male frog occasionally showed strong expression of GFP in trunk muscles (compare Figure 2e with 2e, arrows) but no labelling in the hindlimbs (compare Figure 2e with 2e, arrowheads). Labelling was very dispersed close to the tail tip (Figure 2f), like in "CreER" transgenics, however, the middle of the tail was reasonably labelled (Figure 2g). The inability to label limb muscle with these constructs precluded us from studying limb muscle dedifferentiation by *in vivo *cell tracing, so we proceeded to study it histologically.

### Tail, but not limb myofibres, show signs of dedifferentiation after amputation

**Figure 3:**
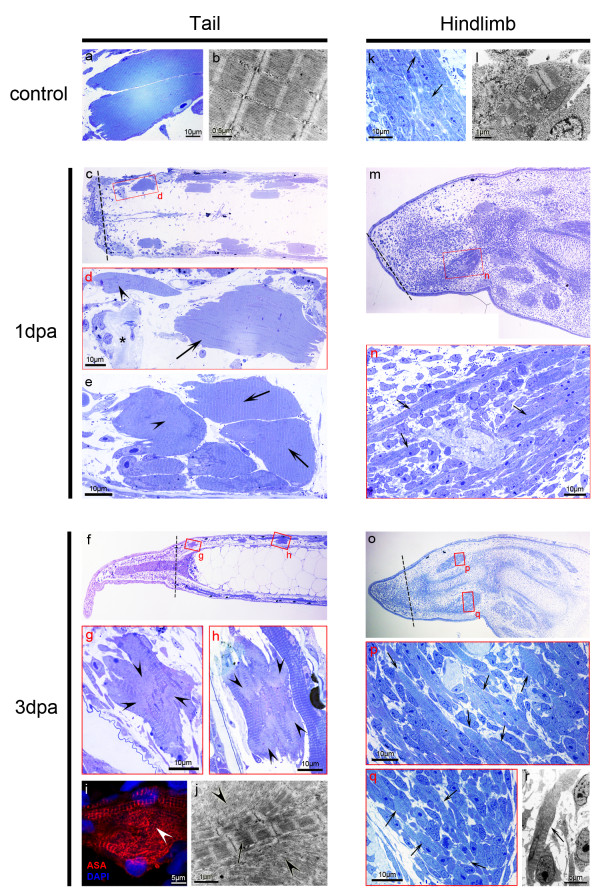
**Tadpole tail myofibres show a dedifferentiation phenotype**. (**a**, **b**) Control tail myofibres, showing the orderly aligned sarcomeric structure in semithin sections (**a**) and TEM micrographs (b). (**c**-**e**) At 1 dpa, dead myofibres are frequently observed next to the amputation plane in the tail (asterisk). Many myofibres show a more compact shape with an apparently normal sarcomeric structure (arrows) while others show some sarcomeric disorganization (arrowhead in *d*). In few cases, the sarcomeric disorganization is more severe (arrowhead in *e*). (**f**-**h**) At 3 dpa, a considerable regenerate has formed (**f**) and more stump myofibres show the dedifferentiation phenotype (**g**, **h**). (**g**) Myofibre close to amputation plane with an irregular shape and big regions in the sarcoplasm devoid of sarcomeres (arrowhead). (**h**) Another myofibre, farther from the amputation plane, showing the same phenotype (arrowheads). Externally to this myofibre there is a longer fibre. These longer muscle fibres never show an obvious dedifferentiation phenotype. (**i**) Confocal image of a tail myofibre with disarranged alpha-sarcomeric actin (ASA, arrowhead) at 3 dpa. (**j**) TEM micrograph of a myofibre with dissociating myofibrils (arrowheads) next to a region with organized myofibrils (arrow), at 3 dpa. (**k**, **l**) Muscle in the zeugopod of stage 54 hindlimbs is very immature. It is mainly composed of myoblasts and young myofibres (arrows). (**m**, **n**) At 1 dpa, no obvious changes are observed (arrows: young myofibres). (**o**-**r**) At 3 dpa, distal myofibres are still very similar to the ones observed at 0 and 1 dpa (arrows: young myofibres). No obvious dedifferentiation was observed in the limb muscle. Dashed lines: amputation planes. *n *≥ six sections per animal, three animals per time point.

One of our main goals in this study was to detect the presence or absence of muscle dedifferentiation during tadpole limb regeneration. Since we could not obtain results with the cell tracing experiments, we decided to study limb muscle regeneration histologically. It has been known for a long time that *Xenopus *limb regeneration capacity decreases during development: an immature limb bud regenerates perfectly, while a fully differentiated limb does not [37]. Therefore, we analysed stage 54 limbs (staging according to Nieuwkoop & Faber [38]), which lay halfway in between the two extremes, i.e., they are not too immature and still regenerate to some extent. Tail amputations were used as a negative control for muscle dedifferentiation, as it was shown by Gargioli & Slack that tail muscle fibres do not contribute to the regenerate [8]. Surprisingly, we observed a dedifferentiation phenotype in the tail (Figure 3a-j), mainly at 3 days post-amputation (dpa). 0 dpa control tails showed the expected sarcomeric striations occupying basically all the sarcoplasm of the myofibres (Figure 3a, b). At 1 dpa, many myofibres had a less elongated shape with fairly regular sarcomeric striations (Figure 3d, e, arrows) while some fibres had small regions without sarcomeres (Figure 3d, arrowhead) and only in rare cases did myofibres have severe sarcomeric loss (Figure 3e, arrowhead). At 3 dpa, many more stump myofibres showed loss of sarcomeric organization and had irregular shapes, both close and farther from the amputation plane (Figure 3f-h, arrowheads). Alpha sarcomeric actin (ASA) staining also showed evidence of a lack of sarcomeric organization (Figure 3i, arrowhead). Electron microscopy observations showed that myofibrils (Figure 3j, arrow) were detaching from each other, leading to the loss of sarcomeric striations (Figure 3j, arrowheads), a phenotype previously described as dedifferentiation [19,20,39-41].

In the limb the situation was less clear (Figure 3k-r). Stage 54 hindlimbs have very immature distal muscle, grouped in small patches and composed mainly of myoblasts and small myofibres. These small and thin myofibres have few myofibrils and sarcomeres inside their cytoplasm (Figure 3k, l, arrows). At 1 dpa, the myofibres continued to present aligned sarcomeres with no signs of dedifferentiation (Figure 3m, n, arrows). At 3 dpa, the distal muscle patches were still very immature and similar to the ones identified at 0 and 1 dpa. We continued to observe small myofibres with normal striations and no obvious dedifferentiation phenotype (Figure 3o-r), but we recognize that due to tissue immaturity, sarcomeric disorganization might be hard to identify. To obtain more information on limb and tail muscle regeneration, we performed gene expression analysis by quantitative Real Time PCR.

### Tail muscle dedifferentiation is corroborated by gene expression analysis

**Figure 4:**
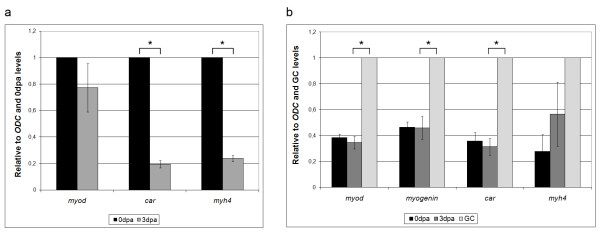
**Expression of mature muscle genes decreases with regeneration in tail and limb**. (**a**) Real Time PCR analysis of tail regeneration. *myod *gene expression does not significantly change in the distal stump between 0 and 3 dpa, while cardiac α actin (*car*) and myosin heavy chain 4 (*myh4*) have 5-fold lower expression at 3 dpa. *n *= three independent experiments of 10 tail samples per time point. (**b**) Real Time PCR analysis of stage 54 limb regeneration. Three samples were used: 0 dpa zeugopod; 3 dpa zeugopod; growth control (GC)-not amputated, three days older zeugopod. *myod, myogenin *and *car *levels are the same between 0 and 3 dpa. However, comparing 3 dpa with GC, we observed a significant lower expression of *myod, myogenin *and *car *in the regenerating limb. *n *= four independent experiments of 10 or 20 limb samples per time point. Results were normalized for ornithine decarboxylase (*odc*) expression and relativized to the time points with highest expression (0 dpa for tail and GC for limb). Error bars: standard error. Asterisk: *p *value < 0.01; Student's *t *test.

During myogenesis, the expression of muscle genes changes from early or progenitor markers (*myod *and *myf5*) to intermediate or differentiation markers (*myogenin *and *MRF4*) and finally to late or structural markers (actins, myosins and others) [42,43]. However, during muscle dedifferentiation, the expression of many muscle genes, mainly the structural ones, is expected to decrease [28,44-47]. Our Real Time PCR analysis showed that the expression of the late muscle genes *car *(alpha-cardiac actin) and *myh4 *(myosin heavy chain 4) was 5-fold lower in the 3 dpa distal tail stump compared to the 0 dpa distal stump (Figure 4a). This indicates that, after tail amputation, muscle fibres decrease the expression of structural proteins, or, in other words, that the myofibres dedifferentiate. Since we later observed that a considerable number (but no more than half) of myofibres located in the analysed region die after amputation, we repeated the tail amputations but collected 3 dpa stump samples 1 mm away from the amputation plane, a region with negligible fibre loss. With these samples, we observed a downregulation of *car *to 53.7% ± 4.5% (standard error, *p *value = 49 × 10^-5^) compared with 0 dpa, confirming the dedifferentiation of muscle fibres. In fact, we also observed a visual decrease in car promoter activity as the expression of GFP under the Car promoter decreased in a wide region of the distal stump during tail regeneration (not shown).

In the limb the situation was more complex to analyse. Because the stage 54 limb is growing quickly and more muscle is being added every day, we needed a "growth control" to compare with the 3 dpa distal stump. The growth control (GC) came from stage 54 animals that were not amputated on day 0 but were left to grow for three more days, when the proximal half of the zeugopod was collected. Analogous regions were collected for 0 dpa and 3 dpa. Comparing gene expression between 0 dpa and 3 dpa zeugopods, we found no differential expression of *myod, myogenin *and *car *(Figure 4b), indicating the absence of dedifferentiation. The expression of *myh4 *slightly increased, but the change was not statistically significant (*p *value = 0.34). However, when we compared the 3 dpa tissue with the GC, we observed that the regenerating tissue has a 2- to 3-fold lower expression of *myod, myogenin *and *car*, indicating that myogenesis is halted by the amputation.

### *In vivo *tracing confirms that myonuclei do not contribute to the tail regenerate

The observed signs of tail muscle dedifferentiation were unexpected since it was previously shown by Gargioli and colleagues that tail muscle fibres did not contribute to the regenerate [8]. To confirm Gargioli's results, we returned to our inducible Cre transgenics ("CreER" and "Cre fragments", Figure 2) to follow labelled tail muscle during regeneration. As already stated, a common characteristic of the induced "Cre fragments" transgenics (and to a lesser extent in "CreER" transgenics) was that many of the labelled myofibres were concentrated in the trunk, close to the head and hindlimbs (Figure 2e'), while less labelling was found in the middle of the tail (Figure 2g) and little labelling was present close to the tip of the tail (Figure 2b, f). This allowed us to do two kinds of experiments. One was to amputate through the middle of the tail ("proximal amputations"; only in "Cre fragments" transgenics) and follow the overall changes of the group of labelled fibres during regeneration. The other experiment was to amputate closer to the tip, at around 2/3 of the tail ("distal amputations"; in both "CreER" and "Cre fragments" transgenics), and follow, in detail, individually labelled myofibres. The latter experiment is analogous to one performed by Echeverri *et al*. (2001), where they showed that axolotl tail myofibres fragment to proliferative mononuclear cells. Fibre fragmentation (or cellularization) and re-entry into the cell cycle are independent mechanisms expected to conclude the process of muscle dedifferentiation [5,48].

**Figure 5:**
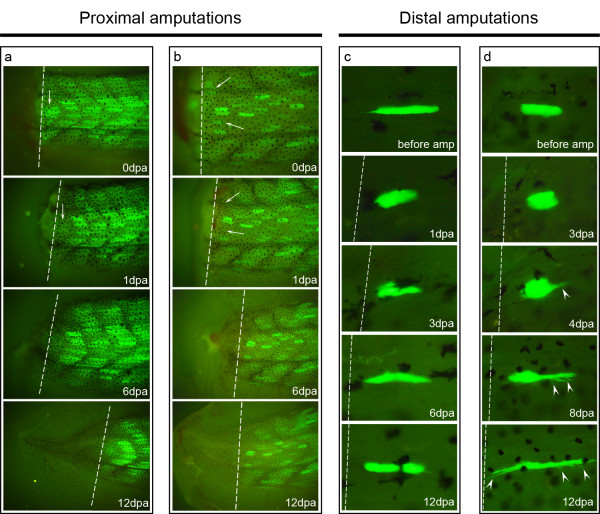
***In vivo *observations show that tadpole tail myofibres do not fragment**. (**a**, **b**) Tail regeneration results of Car-nCre_Hsp-nlcCre_CALNL-GFP F_1 _transgenic tadpoles amputated approximately in the middle of the tail ("proximal amputations"). Many of the labelled myofibres next to the amputation plane were not found at 1 dpa probably because they degenerated. From 1 dpa till the end of the observations, the number and position of labelled myofibres did not change, indicating that there was no myofibre fragmentation. Also, no labelling was observed in the regenerate. *n *= 18 animals. (**c**, **d**) *In vivo *tracing of single myofibres in the distal part of the tail of Car-ERCreER/CALNL-GFP or Car-nCre_Hsp-nlcCre_CALNL-GFP transgenics. Myofibres close to the amputation plane generally showed a compact or retracted shape at 1 dpa (*n *= 94 fibres). During the next days, these muscle fibres started to recover the elongated shape, sometimes forming long citoplasmic projections (arrowheads). At 12 dpa, the shape of the myofibres was again elongated but generally different from the initial shape. Dashed lines: amputation planes.

After proximal amputations of reasonably well labelled tails, we observed that many myofibres located close to the amputation plane were missing at 1 dpa, indicating the death of these fibres (Figure 5a, b, arrows). Treatment of wild-type tadpoles with the vital stain Propidium iodide at 0 dpa showed many positive myonuclei next to the amputation plane, confirming the death of distal-most myofibres (not shown). Over the following days, the number and position of GFP labelled fibres barely changed (please compare 1 dpa with 6 dpa and 12 dpa in both Figure 5a and 5b), indicating that these myofibres were stable and not fragmenting. In addition, no labelling was observed in the tail regenerates after careful examination with higher magnifications. These observations indicate that myofibre cellularization and contribution to the regenerate was not occurring during tail regeneration, confirming Gargioli's results [8].

### Distal myofibres retract after amputation but do not fragment

Taking advantage of the low number of labelled myofibres in the distal part of the tail, we were able to follow individually labelled myofibres during regeneration. A similar experiment done by Echeverri *et al*. in the axolotl tail showed synchronous myofibre fragmentation a few days after the labelled muscle fibre was clipped by the amputation [4]. In our hands, clipping the tip of a myofibre always led to its death (*n *= 14). On the contrary, amputations that did not directly touch the myofibres led to much higher fibre survival. A common characteristic of the surviving myofibres was that they frequently showed a compact or retracted shape at 1 dpa (*n *= 94, Figure 5c, d). In some situations, a slight retraction could already be observed a few minutes after amputation. Between two to four days post-amputation, these myofibres started to recover their size (Figure 5c, d) and sometimes they formed irregular cytoplasmatic projections (Figure 5d, arrowheads). By 12 days post-amputation, these myofibres had generally recovered their initial length (Figure 5c) or were even longer (Figure 5d). So, in contrast to what is described in the axolotl [4], we did not observe myofibre fragmentation in the tadpole tail.

Interestingly, different types of muscle fibres responded differently to amputation: "short" myofibres (around 2/3 the size of a myotome) were much more prone to retract than "long and thin" myofibres (length of a myotome). In fact, all the "short" fibres retracted after distal amputation (*n *= 83, with variable degrees), while only 11 out of 32 "long and thin" fibres slightly retracted after amputation. Therefore, not all the myofibres traced during regeneration showed an obvious retraction phenotype. In several cases, muscle fibres maintained their shape and size from the day of amputation till the end of observations and they generally belonged to three different groups: "long and thin" myofibres (*n *= 21, see example below); fibres from "proximal amputations" (*n *> 100, see below and Figure 5a, b); fibres far from the amputation plane (*n *= 7, not shown).

### Dedifferentiating myofibres do not enter the cell cycle

**Figure 6:**
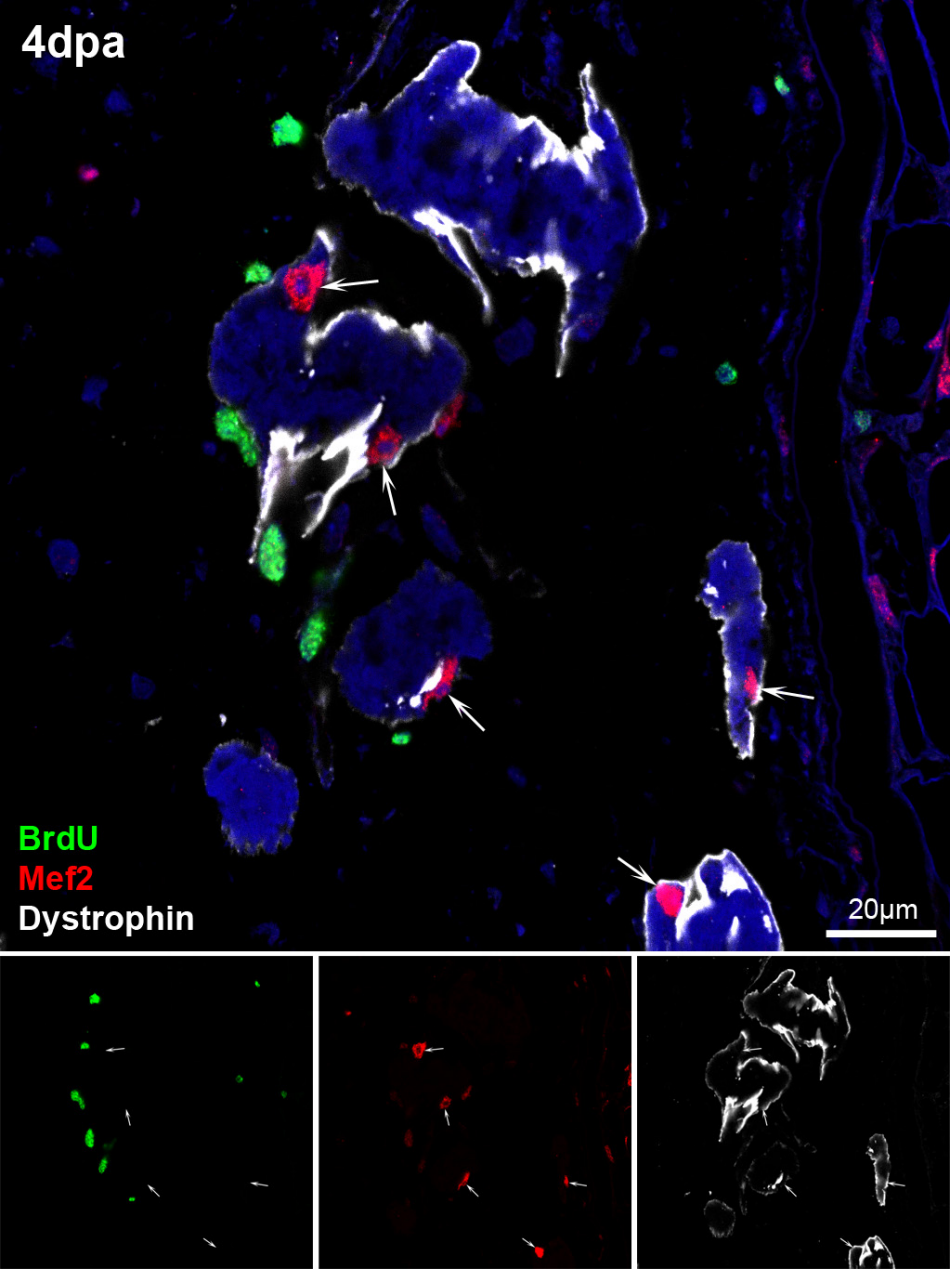
**Dedifferentiating myofibres do not enter the cell cycle**. 4 dpa regenerating tails were fixed 24 hours after intraperitoneal injection of BrdU and immunostained against BrdU (green), Mef2 (red, marking the nuclei of myoblasts and myofibres) and Dystrophin (white, marking the cell membrane of the myofibres). Dedifferentiating myofibres-the ones with irregular shapes-never showed myonuclei positive for BrdU (arrows; *n *= 100 myonuclei (Mef2^+ ^nuclei inside the dystrophin)).

To analyse the extent of dedifferentiation, specifically if dedifferentiating myofibres entered the cell cycle, we did a BrdU pulse to label cells synthesizing DNA. BrdU was injected intraperitoneally at 3 dpa (when dedifferentiation is most frequent) and tail samples were fixed 3, 24 and 72 hours later. All myonuclei were BrdU negative in the samples fixed 3 and 24 hours after injection (Figure 6, arrows), showing that no dedifferentiating myofibre entered S-phase at 3 dpa (*n *= 76 BrdU^- ^myonuclei at 3 h + 100 BrdU^- ^myonuclei at 24 h). On the other hand, many cells were proliferating at 3 dpa and 12% of them were found to be activated satellite cells (Pax7^+^; *n *= 34 BrdU^+^/Pax7^+ ^of 294 BrdU^+ ^nuclei; not shown). As a result, the samples fixed 72 hours after the pulse had many muscle fibres with BrdU positive myonuclei (*n *= 33 BrdU^+ ^myonuclei; not shown). These labelled nuclei most likely derived from satellite cells that were proliferating at 3 dpa and later fused with new or pre-existing myofibres. In addition, PCNA and pHH3 staining confirmed the absence of cell cycle in dedifferentiating myofibres at various days post-amputation (not shown). These results, together with the *in vivo *tracing, indicate that tadpole tail myofibres experience an incomplete dedifferentiation, lacking cell cycle re-entry and fragmentation.

### Dedifferentiation is associated with myofibre retraction

**Figure 7:**
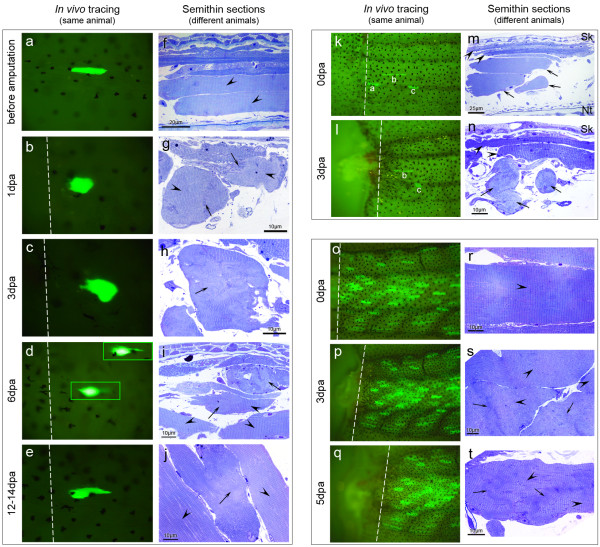
**Relation between myofibre retraction and dedifferentiation phenotype**. (**a**-**j**) During regeneration, "short" myofibres change their shape from elongated (before amputation) to compact (1-4 dpa) and back to elongated (6-12 dpa) (a-e, *e *corresponds to 12 dpa; *n *= 94 fibres). This change in shape is also observed in semithin sections of wild-type animals fixed at different days post amputation (f-j, *j *corresponds to 14 dpa; *n *≥ six sections per animal, three animals per time point). At 1 dpa (**b**, **g**), "short" myofibres are retracted (**b**) and the dedifferentiation phenotype is occasionally observed in fibres with similar compact shapes (g, arrows point to disorganized sarcomeres, arrowheads point to normal sarcomeres. See also Figure 3c-e). At 3 dpa (**c**, **h**), the dedifferentiation phenotype is more common and is generally present in myofibres with irregular shapes (h, arrow), i.e., the ones that retracted (**c**). At 6 dpa (**d**, **i**), retracted myofibres start to have a more elongated shape (d, boxes correspond to different focal planes) and the dedifferentiation phenotype is now less common (i, arrows), with more myofibres showing normal sarcomeres (i, arrowheads). At 12-14 dpa (**e**, **j**), elongated shape is regained (**e**) and many myofibres show a normal internal structure (j, arrowheads), while few still have some sarcomeric disorganization (j, arrow). (**k**-**n**) The "long and thin" myofibres were less prone to retract (compare fibre "b" in *k *and *l; n *= 21 fibres), in contrast to "short" myofibres that become even shorter with amputation (compare fibre "c" in *k *and *l*). Comparatively, "long and thin" myofibres did not show the dedifferentiation phenotype at 3 dpa (arrowheads in *m *and *n*), while short myofibres did (arrows in *m *and *n*). Myofibre "a" died with the amputation (was not found at 1 dpa). Nt: notochord. Sk: skin. (**o**-**t**) Proximal amputations (done approximately at the middle of the tail) generally resulted in no or little myofibre retraction (o-q; *n *= 18 animals). Likewise, semithin sections (**r**-**t**) revealed that myofibres generally kept the organized internal structure (arrowheads), with some myofibres showing only a mild disorganization of the sarcomeric structure (arrows). Dashed lines: amputation planes.

Several reasons led us to believe that the dedifferentiation phenotype observed histologically (Figure 3c-j) was related to the muscle fibre retraction observed *in vivo *(Figure 5c, d). Firstly, the dedifferentiating fibres had irregular and compact shapes that resembled the retracted fibres observed *in vivo *(Figure 7a-j). As described previously, myofibres had an elongated appearance before amputation (Figure 7a) with well organized sarcomeres (Figure 7f, arrowheads). At 1 dpa, many myofibres showed a more compact shape *in vivo *(Figure 7b and 5c, d) as well as histologically, with occasional loss of sarcomeric striations (Figure 7g, arrows. See also Figure 3c-e, arrowheads). At 3 dpa, the retracted fibres still had a compact shape *in vivo *(Figure 7c), but now, a more frequent dedifferentiation phenotype was visible in the retracted/irregularly-shaped myofibres, histologically (Figure 7h, arrow). At 6 dpa, the retracted myofibres were recovering their elongated shape *in vivo *(Figure 7d) and, histologically, the myofibres showing signs of dedifferentiation also had a more elongated shape (Figure 7i, arrows). The dedifferentiation phenotype was now less frequent, since more muscle fibres had normal sarcomeric striations (Figure 7i, arrowheads). Later in regeneration, the previously retracted myofibres regained an elongated shape (Figure 7e and 5c, d (at 12 dpa)) and only rare dedifferentiation remnants were observed histologically (Figure 7j, arrow (at 14 dpa)). The majority of myofibres close to the amputation plane showed normal sarcomeric striations at 14 dpa (Figure 7j, arrowheads).

Secondly, retraction and dedifferentiation phenotypes were specific to one type of myofibres. Sasaki described that the tadpole tail has two types of muscle fibres: "red" fibres, with a small diameter and located externally, very close to the skin (Figure 7m, arrowheads); and "white" fibres, which are wider and located internally, below the red fibres (Figure 7m, arrows) [49]. By shape and size comparison, we concluded that the "short" fibres observed *in vivo *(Figure 7k, fibres "a" and "c") are white (internal) fibres, while the "long and thin" (Figure 7k, fibre "b") are red (external) fibres. These myofibres behaved differently after distal amputation, since the short and internal myofibres always retracted after amputation (Figure 7k, l; compare size and shape of fibre "c" between 0 dpa and 3 dpa) and showed the dedifferentiation phenotype at 3 dpa (Figure 7n, arrows). In contrast, the long and thin myofibres, generally, did not contract (Figure 7k, l. Compare fibre "b" between 0 dpa and 3 dpa) and never showed the histological dedifferentiation phenotype after amputation (Figure 7n, arrowheads).

Finally, we also observed a relationship between retraction and dedifferentiation when we compared proximal and distal amputations. Although retraction was commonly observed after distal amputations, it was much less frequent and noticeable after proximal amputations (Figure 7o-q and Figure 5a, b). Accordingly, semithin sections revealed that myofibres frequently showed regular sarcomeric structure after proximal amputations (Figure 7s, t, arrowheads) with some myofibres showing very small regions of sarcomeric disorganisation (Figure 7s, t, arrows).

### Larval zebrafish myofibres do not dedifferentiate after tail amputation

**Figure 8:**
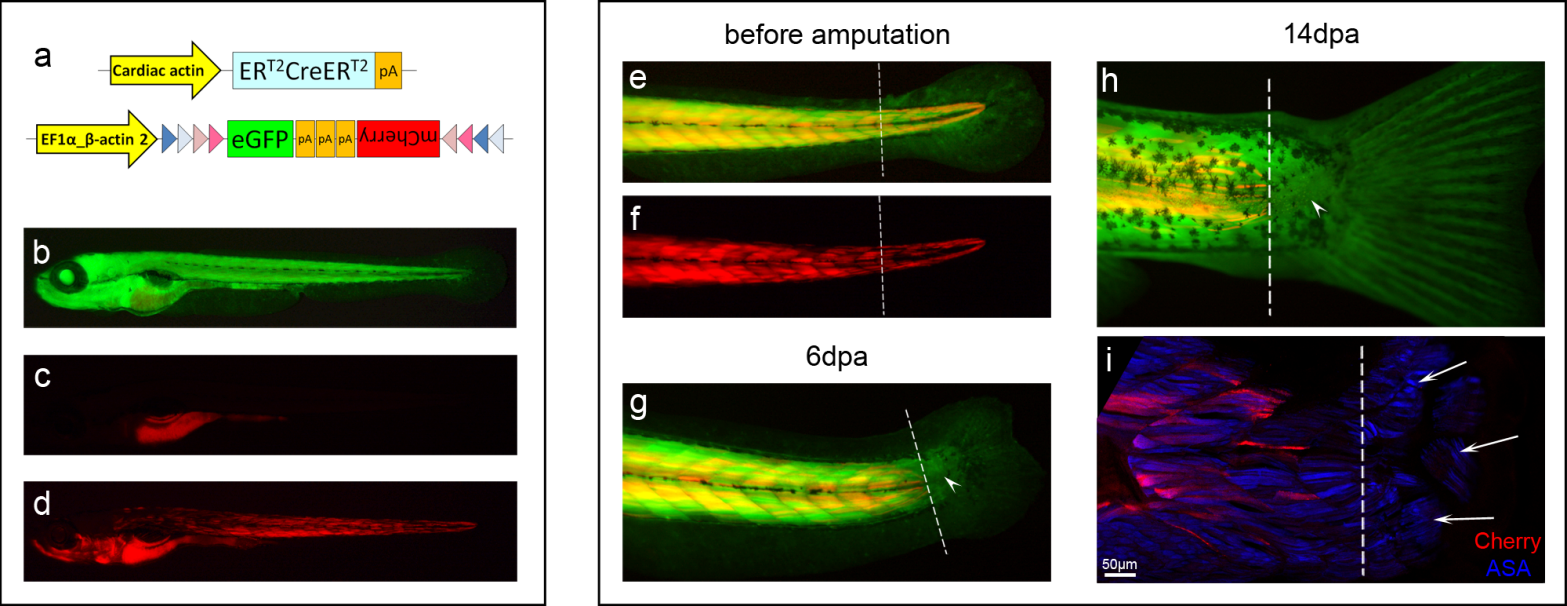
**Labelled myofibres do not contribute to new muscle during regeneration of zebrafish larvae tail**. (**a**) Representation of the Car-ERCreER and eab2-EGFPTmCherry [52] constructs, which allow the expression of mCherry in muscle fibres after activation of Cre. (**b**) These transgenics expressed eGFP ubiquitously. (**c**) Without tamoxifen treatment, mCherry expression was not observed (gut is autofluorescent). (**d**) After tamoxifen treatment, mCherry was generally visible in many muscle fibres. (**e**, **f**) Tails of two weeks old zebrafish larvae were amputated proximal to the base of the fin fold. (**g**) Six days later, some animals had regenerated a small piece of tail (arrowhead) and a caudal fin. (**h**) At 14 dpa, the small tail regenerate (arrowhead) continued to be free of mCherry labelling. (**i**) Confocal image of the same region, showing that the small tail regenerate has muscle fibres (arrows, ASA: α-sarcomeric actin) that are not labelled with mCherry. Pictures *e *to *i *correspond to the same animal. Dashed line: amputation plane. *n *= 33.

Very little is known about skeletal muscle regeneration in fish. Recent reports have shown that dedifferentiation is very common during regeneration of zebrafish tissues [6,40,50,51], but it remains unknown if this is true for skeletal muscle regeneration. To study muscle regeneration in the zebrafish, we cloned the Car-ER^T2^CreER^T2 ^construct (without the reporter part) into a Tol2 vector and made transgenics in a *Tg(eab2:[EGFPTmCherry]) *background [52] (Figure 8a). These transgenics ubiquitously expressed GFP (Figure 8b), but no mCherry (red fluorescence) (Figure 8c). When tamoxifen was added, Cre became active and GFP was switched for mCherry expression specifically in muscle fibres (Figure 8d).

For the regeneration studies, we treated embryos with tamoxifen and amputated two week old larvae proximally to the base of the fin fold, removing about 1/5 of the tail musculature (Figure 8e, f). Six days after amputation, we could observe a small tail regenerate proximal to the regenerated caudal fin, generally smaller than the length of two myotomes, without mCherry labelling (Figure 8g, arrowhead). Although the new caudal fins continued to grow with time, the small tail regenerates stayed short in length and free of mCherry labelling (Figure 8h, arrowhead (14 dpa)). We further confirmed that the regenerated tail indeed contained new muscle (Figure 8i). The absence of mCherry labelling in the regenerate indicated that the new muscle did not derive from dedifferentiated stump fibres. Although the number of mCherry^+ ^fibres stayed approximately the same during regeneration, we observed that *de novo *myogenesis occurring in the stump (due to the overall growth of the animal) was depositing new GFP^+^/mCherry^- ^fibres next to the old mCherry^+ ^ones. This led to a dispersion of mCherry^+ ^fibres, resulting in stumps with many GFP^+ ^fibres and a few mCherry^+ ^fibres at 21-28 dpa, impeding clear and illustrative pictures at these later time points.

**Figure 9:**
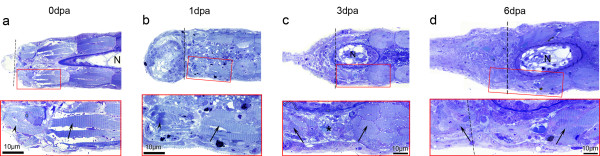
**Zebrafish larvae show no muscle dedifferentiation phenotype**. Micrographs from semithin sections showed that larval tail myofibres have a well organized sarcomeric structure that do not show signs of dedifferentiation during regeneration (arrows). (**a**, **b**) At 0 dpa and 1 dpa, distal most fibres show altered sarcomeric striations, characteristic of damaged or dying myofibre (arrowheads). (**c**) At 3 dpa this phenotype was not observed (neither at 2 dpa, not shown), instead, less muscle is observed close to the amputation plane (asterisk). (**d**) At 6 dpa, new myofibres are visible in the regenerate (left arrow). Notochord (N) is visible as an oval at 3 and 6 dpa because of the slight dorsal curvature that regenerating tails acquire (see Figure 8h). Dashed lines: amputation planes. *n *≥ four sections per animal, three animals per time point.

The absence of muscle dedifferentiation was corroborated by histological observations. At 0 dpa and 1 dpa, we observed that the myofibres closest to the amputation plane showed a damaged phenotype, characterized by the alteration and darkening of the sarcomeric striations (Figure 9a, b, arrowheads). A similar phenotype was observed in *Xenopus *tail myofibres damaged by the amputation, at 0 dpa (not shown) [53]. The remaining fibres, although much smaller than the *Xenopus *tail myofibres, showed clear and regular sarcomeric striations (Figure 9a, b, arrows). At 3 dpa (and 2 dpa, not shown) damaged fibres were no longer visible. Instead, a region with few muscle fibres was present, implying that the majority of damaged fibres had degenerated (Figure 9c, asterisk). Moreover, we did not observe signs of myofibre dedifferentiation. Between 4 and 5 dpa no significant changes were observed (not shown) and at 6 dpa we could confirm that the small regenerate was being populated by new muscle cells (Figure 9d). These results, together with the cell tracing experiments, indicate that muscle dedifferentiation does not occur during zebrafish skeletal muscle regeneration.

## Discussion

Our results indicate that skeletal muscle dedifferentiation is less common than previously believed. In both the *Xenopus *tadpole hindlimb and zebrafish larvae tail we found no evidence of myofibre dedifferentiation after amputation. In addition, we confirmed previous results showing that tadpole tail muscle did not contribute to the regenerated tissues, although we observed downregulation of muscle genes and sarcomeric disorganization in stump myofibres, features characteristic of the onset of dedifferentiation. Nevertheless, we did not observe myonuclei entering the S-phase of the cell cycle, nor myofibres undergoing fragmentation, and therefore consider that the dedifferentiation of tadpole tail muscle is incomplete. This incomplete dedifferentiation strongly suggests that tadpole satellite cells and other muscle progenitors are enough to form the regenerated muscle, and maybe, muscle progenitor cells in the salamanders are not as efficient or present in sufficient numbers, so that old myofibres have to fragment and contribute to the new muscle.

*In vivo *labelling of tadpole tail myofibres showed that a very common type of muscle fibres (white fibres) drastically retract their shape soon after amputation. During the following days we observed how these retracted myofibres regained their elongated shape, indicating that they would recover their function. Histological analysis of the same type of fibres evidenced the loss of shape and of sarcomeric striations (dedifferentiation) in the first days after amputation and the apparent recovery of the elongated shape and striations later on in the regenerative process. Although by histology alone we could not be sure that the dedifferentiated fibres would survive, our *in vivo *studies proved unambiguously that these fibres recovered from those initial drastic changes. Thus it seems that it is more profitable to the animal to recover these fibres rather than inducing their death, probably for energetic and/or timing reasons.

Despite muscle dedifferentiation in the tadpole tail seems to be fruitless, it could be necessary for muscle regeneration, for instance, for satellite cell activation as Morrison *et al*. suggested [10]. However, dedifferentiation is almost absent after proximal amputations, indicating that it is not needed for regeneration or satellite cell activation, since proximal amputations also regenerate the muscle. The observation that dedifferentiation is rare after proximal amputations also indicates that the processes involved in the onset of regeneration, for example, bioelectric fields, coagulation, inflammation and signals from the wound epidermis [54-56], are not responsible for the observed muscle dedifferentiation. In fact, myofibre retraction is the only process found to be associated with the histological dedifferentiation phenotype.

Tadpole tail muscle dedifferentiation reaches its peak at 3 dpa, when sarcomeric loss is most intense and the expression of mature muscle genes has been reduced 2- to 5-fold. However, some retracted myofibres already show loss of sarcomeres at 1 dpa. This suggests that the mechanical pressure from tissue retraction can passively cause some disarrangement of myofibrils. Nevertheless, the observation of more frequent sarcomeric loss at 3 dpa together with downregulation of muscle genes, strongly indicates the presence of an active process responsible for the dedifferentiation phenotype. It is probable that the initial damage caused by retraction results in the activation of signalling pathways that lead to degradation of muscle proteins (see [57]) and to downregulation of muscle genes. Similar pathways might be present during muscle regeneration in urodeles, since myofibre damage was shown to be necessary for dedifferentiation in the axolotl [4]. The reason why tadpole muscle fibres do not complete the dedifferentiation process might be due to the lack of some effectors or branches of these pathways, for example, the ones involved in cell cycle activation of the myonuclei.

The study of limb muscle regeneration was hampered by the inability to label muscle fibres with the inducible constructs, probably because ER^T2^-Cre-ER^T2 ^induction and/or activity is very weak in the tadpole. Previous reports have shown that regular Cre recombinase is not able to efficiently tag every muscle fibre in the tadpole [8,58] while ER^T2^-Cre-ER^T2 ^appears to be three times less active than regular Cre [31], which explains, to some extent, our failure to label fibres efficiently. However, to exclude the possibility that the "Car-ERCreER/CALNL-GFP" construct had some intrinsic problems, we injected it in zebrafish eggs. After a short treatment with tamoxifen, we observed mosaic GFP expression in muscle fibres, indicating that the construct was working as expected (not shown). This led us to assume that the double ER^T2 ^might be more stringent in tadpoles and/or that tamoxifen could not efficiently reach muscle fibres. In fact, we tried very long treatments with the highest tamoxifen concentrations we could, with no improvements. The use of "Cre fragments" transgenics allowed better labelling in the trunk and anterior tail but still no labelling was observed in the limbs, and very little in the posterior tail. Myogenesis in the limb and posterior tail is initiated later than in the anterior tail [58,59], indicating that later born fibres are much more difficult to label. These difficulties prompted us to study hindlimb regeneration histologically, which revealed no signs of muscle dedifferentiation, contrary to what we observed after tadpole tail amputation. Gene expression analysis during limb regeneration denoted a possible delay of muscle growth after amputation, since the total amount of muscle did not change after three days of regeneration, while it roughly tripled during the same period when the limbs were not amputated. The intense myogenesis occurring in the limbs, could mask the gene downregulation from a possible dedifferentiation, however, we did not find signs of dedifferentiation through histological analysis.

The results obtained on skeletal muscle regeneration of zebrafish larvae were also surprising since several tissues in fish have been shown to regenerate by dedifferentiation, including cardiomyocytes [6,40,41,50,51]. We did not observe signs of muscle dedifferentiation after tail amputation, both by *in vivo *cell tracing and histological analysis, indicating that muscle dedifferentiation is less common than previously believed. However, working with larvae proved to be limiting in regards to obtaining illustrative pictures from late regeneration stages, since during larval growth, many new myofibres are deposited next to the fibres labelled before amputation, which resulted in a considerable dispersion of the labelled fibres. To solve this issue, juvenile or adult animals could be used, but ethical and technical restraints were prohibitive.

To clarify the strategy or mechanism of skeletal muscle regeneration in urodeles, similar inducible cell tracing experiments are needed to definitively confirm that stump myofibres contribute to the regenerated tissues. Future effort should also go into quantifying the contribution of satellite cells (or other muscle progenitors) versus dedifferentiated muscle fragments towards the new muscle. We believe that this contribution might vary between species and between developmental stages within the same species. For example, larval urodeles were shown to have satellite cells inside the myofibre basal lamina, like mammals and frogs, however, in adult urodeles, satellite cells are surrounded by their own basal laminae [7,60] and this can result in satellite cells with different regeneration capabilities.

## Conclusions

Cell tracing and/or histological studies did not reveal any signs of muscle dedifferentiation after tadpole limb or zebrafish tail amputation. However, we detected the unexpected presence of muscle dedifferentiation during tadpole tail regeneration. Nevertheless, dedifferentiation was incomplete since it did not result in myofibre fragmentation or cellularization, nor in cell cycle re-entry, which raises some doubts on the extent of the dedifferentiation phenotypes described histologically in other amphibians. Our results also highlight the importance of using *in vivo *tracing experiments in conjunction with histology to obtain clearer evidence on the events occurring during regeneration.

## Methods

### DNA constructs

For muscle specific expression of GFP in tadpoles, we used the vector pCarGFP2 (Figure 1a), a kind gift from E. Amaya. For the tamoxifen-inducible expression of Cre (and GFP) in muscle cells, the Car-ERCreER/CALNL-GFP vector (Figure 2a) was built using the commercially available multisite Gateway system (Invitrogen), as described by Kwan *et al*. [61]. Before that, a new destination vector containing I-SceI restriction sites flanking the *att *sites was made, for the easy removal of the vector backbone before transgenesis. For that the 1780 bp XhoI/ClaI fragment of pDestTol2pA2 [61] was cloned into I-SceI-pBSII_SK + [62]. To build the 5' entry clone (p5E_Car), the 3500 bp Asp718/SalI fragment from pCarGFP2 was cloned into p5E-MCS [61]. To make the middle entry clone (pME_ERCreER-pA) the 3500 bp ER^T2^CreER^T2^-pA fragment was amplified from pCAG-ER^T2^CreER^T2 ^vector (Addgene plasmid 13777) [32] with the primers 5'-GGGGACAAGTTTGTACAAAAAAGCAGGCTAGAATTCCCGGGTGAGCC-3' and 5'-GGGGACCACTTTGTACAAGAAAGCTGGGTAAGCTTGGGCTGCAGGTC-3' (vector sequences underlined). The 3' entry clone was initially made by amplification of the 4400 bp CAG-lox-neo-lox-GFP-pA fragment from pCALNL-GFP vector (Addgene plasmid 13770) [32] with the primers 5'-GGGGACAGCTTTCTTGTACAAAGTGGATTACGCCAAGCTT*GGG*C-3' and 5'-GGGGACAACTTTGTATAATAAAGTTGTTCCCCGAAAAGTGCCAC-3', but then the Rabbit globin poly(A) sequence was substituted by the SV40 poly(A) sequence. All the clones were confirmed by restriction mapping and sequencing.

For the Heat-shock-inducible expression of Cre in tadpole muscle cells, two different vectors were built (Figure 2d), both constructed with the Gateway system. One vector was the Car-nCre, where N-terminal Cre fragment was cloned under the control of Car promoter. This Cre fragment corresponds to the first 190 amino acids of Cre protein fused with the "N-peptide" used by Xu *et al*. [35]. The corresponding 700 bp sequence was amplified using the template from pCAG-ER^T2^CreER^T2 ^vector and the following mix of primers: 5'-GGGGACAAGTTTGTACAAAAAAGCAGGCTACCATGGCACCCAAGAAGAAGAG-3'; 5'-ATGGCACCCAAGAAGAAGAGGAAGGTCGACAATTTACTGACCGTACAC-3'; 5'-GGGGACCACTTTGTACAAGAAAGCTGGGTTCATTGAGCCAGCTCCTTCTTC-3'; 5'-ATTGAGCCAGCTCCTTCTTCAGAGCTTGGAGTTCCCACTTCAGCTGAGC-3'; 5'-GTTCCCACTTCAGCTGAGCCAGTTCCTTCTTATTAGCTTGGAGCTCCTTCTTCAG-3'; 5'-GCTTGGAGCTCCTTCTTCAGGGCACCAGAACCACCGTCAGTACGTGAGATATC-3'. The amplification product was then used to make the middle entry clone pME_nCre. Gateway LR reaction was made with p5E_Car, pME_nCre and p3E_polyA [61]. The second vector was the Hsp-nlcCre, where C-terminal Cre fragment was cloned under the control of Hsp70 promoter [63,64]. This Cre fragment corresponds to the last 154 amino acids of Cre protein fused to a nuclear localization signal and the "C-peptide" used by Xu *et al*. [35]. The corresponding 650 bp sequence was amplified from pCAG-ER^T2^CreER^T2 ^vector with the following mix of primers: 5'-GGGGACAAGTTTGTACAAAAAAGCAGGCTACCATGGCACCCAAGAAGAAGAG-3'; 5'-ATGGCACCCAAGAAGAAGAGGAAGGTGTCCGAACAACTGGAGAAGAAGCTCCAG-3'; 5'-CAACTGGAGAAGAAGCTCCAGGCTCTCGAAAAGAAGCTGGCTCAACTCGAATG-3'; 5'-AAGCTGGCTCAACTCGAATGGAAGAATCAAGCTCTGGAAAAGAAGCTCGCCCAAG-3'; 5'-GAAAAGAAGCTCGCCCAAGGCTCTGGTGGGAGAATGTTAATCCATATTG-3'; 5'-GGGGACCACTTTGTACAAGAAAGCTGGGTCTAATCGCCATCTTCCAGCAG-3'. The amplification product was used to make the middle entry clone pME_nlcCre. For the 5' entry clone (p5E_Hsp70), the Hsp70 promoter sequence was extracted from HGEM vector [64] with KpnI and BamHI and cloned it into p5E-MCS [61]. Gateway LR reaction was made with p5E_Hsp70, pME_nlcCre, p3E_polyA [61] and the I-SceI destination vector.

For the tamoxifen-inducible expression of Cre in Zebrafish muscle fibres, the Tol2_Car-ERCreER vector was built using the Gateway system. The 5' entry clone used was p5E_Car (see above), while a new middle entry clone (pME_ERCreER, without poly(A)) was made from pCAG-ER^T2^CreER^T2 ^vector. For this PCR we used the same Fwd primer as before and a new Rev: 5'-GGGGACCACTTTGTACAAGAAAGCTGGGTCCGCTATCAAGCTGTGGCAG-3'. The 3' entry clone (p3E_RgpA) was obtained by amplification of the 550 bp Rabbit beta-globin poly(A) sequence from pCAG-ER^T2^CreER^T2 ^vector, with the primers 5'-GGGGACAGCTTTCTTGTACAAAGTGGCGCACTCCTCAGGTGCAG-3' and 5'-GGGGACAACTTTGTATAATAAAGTTGCAAGCTTGGGCTGCAGGT-3'. LR reaction was made with the pDestTol2pA2 destination vector.

### Transgenesis

*Xenopus *transgenesis was performed as described in Smith *et al*. [65] with the following modifications: egg collection was done right before mixing the sperm nuclei with the linearized DNA(s); eggs were dejellied in 2.5% L-cysteine hydrochloride in 1x MMR (no Ca^2+^) pH 8,5 (as in [66]), washed in 1x MMR (no Ca^2+^) + 0.5% BSA and plated with injection solution supplemented with 0.5% BSA; Sperm nuclei + DNA was diluted in 200 ul of MOH (as in [66]) before injection; 4 or more hours after injection, cleaving embryos were transferred to culture medium (0.3x MMR + 0.5% BSA + 0.05% gentamycin) [66] and incubated overnight at 17°C; after gastrulation, embryos were transferred to 0.1x MMR + 0.05% gentamycin.

The DNA(s) were prepared as follows: pCar-GFP was linearized with NotI; Car-ERCreER/CALNL-GFP was digested with I-SceI and separated from the backbone by gel electrophoresis; pCALNL-GFP was linearized with ScaI while Car-nCre and Hsp-nlcCre were digested with I-SceI and separated from the backbone by gel electrophoresis. Each DNA was used at a concentration ranging from 50 fmol to 200 fmol.

For zebrafish transgenesis, 2 ul of Tol2_Car-ERCreER DNA (50 ng/ul) was mixed with 2 ul of Tol2 transposase RNA (40 ng/ul) and injected into one-cell-stage embryos derived from the cross between a wildtype (AB Salk) female and a *Tg(eab2:[EGFP-TmCherry]) *male [52].

### Treatments for the induction of Cre recombinase

For the activation of tamoxifen-inducible Cre (ER^T2^CreER^T2^) in *Xenopus *tadpoles, we did daily intraperitoneal injections of 4-Hydroxytamoxifen (4-HT) during two weeks. 4-HT was dissolved in ethanol at a concentration of 50 mM or 10 mM and then freshly diluted 1/25 or 1/10 (to 2 mM) in water, before the injections. Injections were started when animals were 2 weeks old. For the activation of heat-shock-inducible Cre (nCre + nlcCre) in *Xenopus *tadpoles, we did daily heat-shocks for two weeks. Heat-shocks were done at 34°C for 20 minutes in tempered 0.1x MMR. Heat-shocks were initiated when tadpoles were between 10 and 20 days of age. Both 4-HT injected and heat-shocked animals had a resting period of 4 to 7 days before amputation.

For the activation of tamoxifen-inducible Cre in zebrafish larvae, we did treatments of 2 μM 4-HT in embryo medium [67] between 1 to 4 days post-fertilization.

### BrdU and Propidium iodide treatments

For the detection of cycling nuclei, approximately 30 μl of BrdU (Sigma, 16880) dissolved in water at 2 μg/μl was injected intraperitoneally into stage 53 tadpoles 3 days after tail amputation. Samples were collected 3, 24 and 72 hours later.

For the detection of dying nuclei *in vivo*, we injected intraperitoneally approximately 30 μl of Propidium iodide (Invitrogen, P21493) dissolved in water at 1 μg/μl, into stage 56 tadpoles right after amputation, and observed the results 3 hours later.

### *In vivo *imaging

*In vivo *cell tracing observations were done with Leica MZ16F Fluorescence Stereomicroscope equipped with FluoCombi III, which allows high resolution imaging. Before any manipulation, *Xenopus *tadpoles and zebrafish larvae were anesthetized in tricaine.

### Amputations

Before amputation, the animals were anesthetized in tricaine. Tadpole tail amputations for the cell tracing experiments were done at two levels: around 2/3 of the tail, depending on the position of the labelled myofibres, in what we called "distal amputations"; at the middle of the tail ("proximal amputations"). Animals were 4 to 6 weeks old (stage 53-56). Tail amputations for the Real Time PCRs were done exactly at 2/3 of the tail of stages 49, 50 and 54 tadpoles (10 tadpoles per time point and stage). For the histological observations, tails were amputated at 2/3 or at the middle of stage 53 or 56, respectively (3 tadpoles per time point and stage).

Tadpole hindlimb amputations were done at stage 54 through the middle of the zeugopod, for the Real Time PCRs (10 or 20 limbs per time point, in quadruplicate). For the histological observations, hindlimbs were amputated through the base of the autopod (3 limbs per time point).

Zebrafish tail amputations were done in two week old larvae, at the base of the caudal fin fold (4/5 the size of the tail, excluding the fin). After amputation, the animals were returned to system water.

### RNA extraction, cDNA synthesis and Real Time PCR

The distal tips of *Xenopus *tail stumps (excluding the blastemas), measuring approximately 0,7 mm in length, were collected at 0 and 3 dpa for RNA extraction. Another region, also with 0,7 mm in length but starting approximately 1 mm from the amputation plane to avoid dead fibres, was also collected at 3 dpa. In the case of the hindlimbs, the remaining proximal half of the zeugopod was collected. Samples were generally kept in RNAlater Solution (Ambion) until use. Total RNA was extracted from tissue samples using TRIZOL^® ^method according to the manufacturer's guidelines (Invitrogen) followed by DNaseI RNase-free treatment. RNA concentration and quality was measured using NanoDrop spectrophotometer (ND-1000). Between 250 to 1000 ng total RNA was used for reverse transcription with Cloned AMV First-Strand cDNA synthesis kit (Invitrogen), according to the manufacturer's guidelines. cDNA samples were generally diluted 1/200 and used fresh for Real Time PCR. 5 μl of diluted cDNA was mixed with 5 μl of Fwd and Rev primer mix (5 pmol per primer) and 10 μl of SYBR green (qPCR Master Mix, Invitrogen). Gene expression levels were measured with Applied Biosystems 7300 Real-Time PCR System and analysed using the ΔΔ*C*_t _method, as previously described [17]. Pairs of primers used: *odc *Fwd (5'-TCCATTGAGAGCGTAGGACTTG-3'); *odc *Rev (5'-GAGGCTCGCCGGTGAAATA-3'); *myoD *Fwd (5'-CAACCAAAGGCTCCCCAAA-3'); *myoD *Rev (5'-TGGAGGCTCTCTATGTAGCGAAT-3'); *myogenin *Fwd (5'-GGTATGCAAGAGGAAGACGGTTT-3'); *myogenin *Rev (5'-CGCTTTTCCCGCAAGGT-3'); *car *Fwd (5'-ACTATGTGTGACGACGAGGAGACTA-3'); *car *Rev (5'-CCAGCCCGGAGCCATT-3'); *myh4 *Fwd (5'-GTGCGTTGTTTGATTCCCAAT-3'); *myh4 *Rev (5'-GCTGGTGGATGAGGAGATGGT-3').

### Histology (semithin and ultrathin sections)

For ultrastructural analysis, tadpole tail samples were collected including at least 4 myotomes to avoid damaging distal muscle fibres, while limbs were collected at its proximal end. Zebrafish larvae were fixed complete. Samples were fixed in 2.5% glutaraldehyde (in 0.1 M Cacodylic buffer, pH 7.2, with sucrose) for 1 hour at 4°C with agitation, and then washed in Cacodylic buffer. Samples were then post-fixed with osmium tetroxide 2%. After dehydration, the samples were embedded with EPON, and frontal sections were made with an ultramicrotome. Semithin sections were stained with toulidine blue and observed with in an optical microscope (Leica DM6000). The ultrathin sections were counterstained with uranyl acetate and lead citrate and observed in a transmission electron microscope (Jeol 1011). At least three animal samples were observed for each time point: control (0 dpa), 1-6 dpa and 14 dpa.

### Immunohistochemistry

*Xenopus *and zebrafish samples were collected as above, fixed in 4% paraformaldehyde (in PBS) for 2 h at 4°C with agitation and embedded in paraffin. *Xenopus *paraffin sections were 5 μm thick, while, due to the low number of mCherry^+ ^fibres in zebrafish tails, we decided to make 10-30 μm thick sections. Sections were dewaxed and antigen retrieval was made only for *Xenopus *samples with Target Retrieval Solution, pH 9 (Dako) in a pressure cooker. The samples were permeabilized with triton X-100 (0.5%) and donkey serum (3%) in TBS. The incubation of the primary antibodies was done overnight at 4°C, and for the secondary antibodies 2 h at 37°C. Primary antibodies used were anti-alpha sarcomeric actin (Sigma, A2172) at 1/400, anti-Dystrophin (DSHB, MANDRA1 clone 7A10) at 1/3, anti-BrdU (Accurate Chemical, OBT0030) at 1/50, anti-Pax7 (R&D Systems, MAB1675) at 1/25, anti-PCNA (Sigma, P8825) at 1/500, anti-pHH3 (Millipore, 06-570) at 1/100 and anti-RFP (AbCam, ab34771) at 1/100. Confocal microscopy was performed with Leica SP5 or SPE confocals.

### Husbandry

Xenopus tadpoles were raised in tanks with a recirculating water system (Aquatic Habitats) at 22 ± 1°C. Adult and juvenile zebrafish were also maintained in tanks with recirculating water at 28 ± 1°C.

### Animal welfare

All animal experiments were done with the approval of institutional (CEEA-PRBB-http://portal.prbb.org/ciencia/comite_etic) and governmental (Generalitat de Catalunya) ethics committees.

## Abbreviations

GFP: Green fluorescence protein; dpa: Days post amputation; F_0_: Founder; F_1_: First generation; F_2_: Second generation; Car: Alpha-cardiac actin; Hsp70: Heat-shock inducible promoter; *myh4*: Myosin heavy chain 4; "CreER": "Car-ERCreER/CALNL-GFP"; "Cre fragments": "Car-nCre + Hsp-nlcCre + CALNL-GFP"; BrdU: 5-Bromo-2'-deoxyuridine; TEM: Transmission electron microscope.

## Authors' contributions

AMCR participated in design of the study, made the transgenic animals, did the cell tracing experiments, collected the samples for histological studies, performed Real Time PCR and BrdU studies and drafted the manuscript. BC conceived the study, participated in its design, built some DNA constructs, collected samples for histological studies and helped with manuscript writing. MM performed the histological studies and reviewed the manuscript. JCIB participated in design of the study, financial support and reviewed the manuscript. All authors read and approved the final manuscript.
